# Identification of Splicing Regulatory Activity of ATXN1 and Its Associated Domains

**DOI:** 10.3390/biom15060782

**Published:** 2025-05-28

**Authors:** Ai Ohki, Masahide Kato, Yoshitaka Aoki, Arisa Kubokawa, Motoaki Yanaizu, Yoshihiro Kino

**Affiliations:** Department of RNA Pathobiology and Therapeutics, Meiji Pharmaceutical University, 2-522-1, Noshio, Kiyose-shi 204-8588, Tokyo, Japanm-yana@my-pharm.ac.jp (M.Y.)

**Keywords:** ATXN1, spinocerebellar ataxia type 1, alternative splicing, RNA binding

## Abstract

The expansion of the polyglutamine tract in ATXN1 contributes to the pathogenesis of SCA1. ATXN1 functions as a transcriptional regulator that interacts with multiple transcription factors, and transcriptional dysregulation has been observed in SCA1. In addition, splicing dysregulation has been identified in cells derived from SCA1 patients and model mouse tissues. Although ATXN1 binds to RNA and splicing factors, its direct involvement in pre-mRNA splicing remains unclear. Here, we demonstrate that ATXN1 regulates the alternative splicing of several minigenes. Using an *Mbnl1* minigene, we found that neither expansion nor deletion of the polyglutamine tract affected ATXN1-mediated splicing regulation. Deletion analysis revealed that its splicing regulatory activity involves a central region of ATXN1, the AXH domain, and a nuclear localization signal in the C-terminal region. The AXH domain alone failed to exhibit splicing regulatory activity, whereas the central region demonstrated weak but significant splicing regulation. Full regulatory function required at least one of these regions, suggesting their redundant role in splicing modulation. Importantly, we newly identified the central region as mediating RNA binding. These findings suggest a novel role for ATXN1 in alternative splicing, providing new insights into the mechanisms underlying SCA1 pathogenesis.

## 1. Introduction

Spinocerebellar ataxia type 1 (SCA1) is an autosomal dominant disorder characterized by degeneration of the cerebellum, brainstem, and spinal cord, caused by mutations in the *ATXN1* (*Ataxin-1*) gene [1]. ATXN1 contains a polyglutamine (polyQ) tract encoded by a CAG repeat sequence, which normally ranges from 14 to 34 repeats but is expanded to 39 or more in SCA1 [2]. ATXN1 is a transcriptional regulator highly expressed in neurons. Expansion of the polyglutamine tract alters ATXN1’s protein–protein interactions and leads to the formation of nuclear inclusions. Knockout mice for *Atxn1* do not exhibit significant neurodegeneration, whereas transgenic and knock-in mice expressing mutant *ATXN1* recapitulate SCA1-associated neuronal degeneration [3,4,5]. Thus, the primary pathogenic mechanism of SCA1 is thought to be the toxic gain of function of mutant ATXN1. However, the loss of normal ATXN1 function has also been suggested to contribute to SCA1 pathogenesis [6]. In addition to SCA1, ATXN1 has been implicated in various neurological disorders, such as Alzheimer’s disease [7] and multiple sclerosis [8], as well as in cancer [9,10]. Therefore, understanding its molecular properties is critical within the context of a broad range of diseases.

Several functional domains of ATXN1 have been identified that contribute to its normal function and/or disease-related processes. ATXN1 interacts with transcriptional repressors such as Capicua (CIC) and plays a role in transcriptional regulation, with the AXH domain specifically mediating its interaction with CIC [11]. The expansion of polyQ leads to a gain of function of the ATXN1-CIC transcriptional repressor complex, resulting in transcriptional dysregulation in SCA1, and the disruption of this protein complex has been shown to be beneficial [12]. Nuclear-cytoplasmic transport of ATXN1 also contributes to SCA1. ATXN1 contains a nuclear localization signal (NLS) near its C-terminal region. In both transgenic and knock-in (KI) mice with expanded *ATXN1*, introducing a K772T mutation, which disrupts the NLS, reduces nuclear localization of ATXN1 and improves disease phenotypes [13,14]. The phosphorylation of Ser776, adjacent to the NLS of ATXN1, can independently induce neurological phenotypes similar to SCA1, even in the absence of polyglutamine expansion [15]. The phosphorylation of Ser776 promotes the interaction of ATXN1 with RBM17, a splicing factor, highlighting the importance of RNA metabolism in the pathogenesis of SCA1 [16]. Interestingly, ATXN1 preferentially binds to RNA with a poly(rG) sequence over poly(rA), poly(rC), and poly(rU) sequences, mediated by a region corresponding to amino acids 277–767 [17]. Subsequently, the AXH domain, overlapping with this region, was shown to bind RNA [18]. Moreover, cells derived from SCA1 patients and model mouse tissues exhibit abnormalities in splicing regulation [19,20]. Thus, accumulating evidence has linked ATXN1 with pre-mRNA splicing. However, it has not been directly investigated whether ATXN1 functions as a splicing regulator.

In this study, we examined whether ATXN1 regulates alternative splicing using reporter genes. We found that both human and mouse ATXN1 exhibit splicing regulatory activity, independent of polyQ length. We then identified the protein regions of ATXN1 that contribute to its splicing regulatory function. During this study, we discovered a novel protein region that mediates both RNA binding and splicing regulation.

## 2. Materials and Methods

### 2.1. Plasmids

*Mbnl1*, *Clcn1*, and *Actn1* minigenes were previously described [21]. The human ATXN1 construct (EGFP-ATXN1) was generated via two-step PCR. In the first step, ATXN1 was amplified from the Clontech Human Brain cDNA Library (Clontech, Mountain View, CA, USA) using the primer set nest-ATXN1-Fw and nest-ATXN1-Rv. The resulting PCR product was used as a template for the second PCR, which was performed using the primers BglII-ATXN1-Fw and XhoI-ATXN1-Rv. The final PCR product was digested with BglII and XhoI and inserted into the BglII/SalI sites of pEGFP-C1 (Clontech) to produce EGFP-ATXN1. The mouse Atxn1 construct (EGFP-Atxn1) was prepared similarly to the human ATXN1 construct via two-step PCR. In the first step, cDNA from the hippocampus of C57BL/6J mice, obtained from a previous study [22], was amplified using the primer set nest-Atxn1-Fw and nest-Atxn1-Rv. This product was then used as a template for the second step, which was performed using BamHI-Atxn1-Fw and SalI-Atxn1-Rv. An artificial NLS (three copies of SV40 NLS) was previously described [23]. All plasmids were validated by sequencing.

The animal study protocol was approved by the Meiji Pharmaceutical University Committee for Ethics of Experimentation and Animal Care (approval number: 2901, date of approval: 29 May 2024).

### 2.2. Cell Culture

Neuro2a (N2a) cells (#CCL-131, ATCC, Manassas, VA, USA) and HEK293 cells (RCB1637, Riken BRC, Ibaraki, Japan) were cultured in Dulbecco’s modified Eagle’s medium (DMEM, Invitrogen, Waltham, MA, USA) supplemented with 10% heat-inactivated fetal bovine serum (Nichirei Bioscience, Tokyo, Japan) and 1% penicillin/streptomycin (Wako, Osaka, Japan). Cells were maintained at 37 °C in a humidified incubator with 5% CO_2_.

### 2.3. Cellular Splicing Assay

A cellular splicing assay was performed as previously described [24]. The primer sets used in the assay are listed in Supplementary Table S1. PCR products of *Mbnl1* and *Clcn1* were analyzed using 2.5% agarose gels. Some key results of Mbnl1 splicing were also analyzed using 12% polyacrylamide gels (Supplementary Figure S5). The insertion of the SM exon in *Actn1* was detected by digesting the PCR product with NcoI, followed by electrophoresis using 12% polyacrylamide gels. The original gel images are provided in Supplementary Figure S6.

### 2.4. SDS-PAGE and Western Blotting

SDS-PAGE and Western blotting were performed according to previously established methods [25]. The antibodies used in this study are listed in Supplementary Table S2 and the corresponding gel images are shown in Supplementary Figure S6.

### 2.5. Intracellular Localization of ATXN1

Neuro2a cells were seeded in eight-well chamber slides (WATSON, Tokyo, Japan). Each ATXN1 construct (0.12 μg) was transfected using Lipofectamine 2000 (Thermo Fisher Scientific, Waltham, MA, USA). The cells were then fixed with 4% paraformaldehyde (Wako), and nuclei were stained with Hoechst 33342 (DOJINDO, Kumamoto, Japan). After staining, the cells were mounted using a mounting medium (Vector Laboratories, Newark, CA, USA). Fluorescence images were captured using confocal microscopy (AX/AXR; Nikon, Tokyo, Japan).

### 2.6. RNA Immunoprecipitation (RIP)

RIP was performed according to previously described methods [26]. In this study, we used anti-GFP mAb-Magnetic Beads (MBL, Tokyo, Japan) for immunoprecipitation and 20 mM DTT instead of 50 mM 2-mercaptoethanol as a reductant for decrosslinking. Mbnl1 RNA was amplified by RT-PCR using primers Mbnl1-int4-Fw and Mbnl1-int5-Rv (Supplementary Table S1). For RIP-seq analysis, we utilized previously published data (SRX9766718), and HEK293T cells expressing mutant ATXN1 were used [27]. We searched for sequence reads mapped to MBNL1 mRNA or pre-mRNA using NCBI BLAST (version 1.4.0).

### 2.7. RNA Pull-Down Assay

Biotinylated poly(rG) RNA was synthesized by Japan BioService (Saitama, Japan). The binding of biotinylated RNA to magnetic beads (Dynabeads MyOne Streptavidin T1, Invitrogen) was confirmed as follows. First, 15 µL of magnetic beads were mixed with 3 µL of biotinylated RNA (20 µM) and incubated with rotation at 4 °C for 2 h. The concentration of biotinylated RNA remaining in the supernatant was then measured using a Nanodrop 1000 (Thermo Fisher Scientific) to verify its binding to the beads. To prepare cell lysates, N2a cells seeded in 12-well plates were transfected with 0.5 µg of each EGFP-ATXN1 expression construct using Lipofectamine 2000 (Invitrogen). Forty-eight hours after transfection, cells were collected and sonicated in 1× RIPA buffer [50 mM Tris, 150 mM NaCl, 1% Triton X-100, 1× protease inhibitor (cOmplete, Roche, Basel, Switzerland)] to obtain whole-cell lysates. The lysates were subjected to protein quantification using the Pierce BCA protein assay kit (Thermo Fisher Scientific), and protein levels were adjusted to ensure consistency across samples. The whole-cell lysates were diluted 2-fold with binding buffer [10 mM Tris, 2.5 mM MgCl_2_, 0.5% Triton X-100, 1 U/µL RNase inhibitor (Ambion, Austin, TX, USA), and 1× protease inhibitor (cOmplete, Roche)], mixed with Dynabeads MyOne Streptavidin T1, and incubated with rotation at 4 °C for 30 min for pre-clearing. A portion of the lysate after pre-clearing was collected as the input fraction. The remaining lysate was then mixed with biotinylated RNA immobilized on streptavidin beads and incubated with rotation at 4 °C overnight. The beads were washed three times with binding buffer, resuspended in SDS sample buffer, and heat-denatured at 98 °C. The input fraction was also treated with SDS sample buffer and heat-denatured. Western blotting was subsequently performed as described in Section 2.4.

### 2.8. Statistical Analysis

All graphs presented in this article were generated using EZR (ver. 2.8-0). Welch’s *t*-test was applied to comparisons between two groups, while Tukey’s post-hoc test was used following one-way ANOVA for comparisons among three or more groups. The graphs were generated using Microsoft Excel.

## 3. Results

### 3.1. ATXN1 Regulates Alternative Splicing

**Figure 1:**
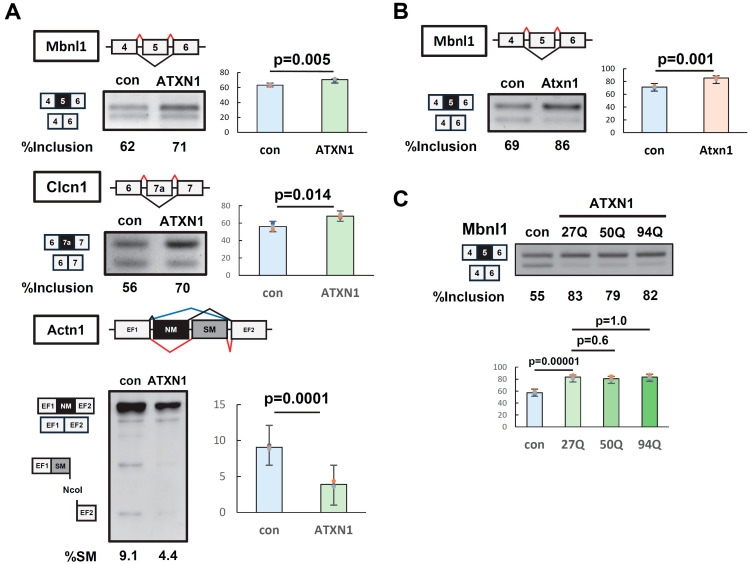
ATXN1 regulates alternative splicing of minigenes. (**A**) Splicing assay of human ATXN1 with a normal polyglutamine tract (27Q) in N2a cells using *Mbnl1*, *Clcn1*, and *Actn1* minigenes. Splice products were detected using reverse transcription–polymerase chain reaction and electrophoresis on either a 2.5% agarose gel or a 12% polyacrylamide gel. To distinguish between the insertion of the NM exon and the SM exon in *Actn1*, the PCR product was digested with NcoI. Black and grey squares indicate alternative exons. (**B**) Splicing assay of mouse Atxn1, conducted as in (**A**). (**C**) Splicing assay of human ATXN1 with normal (27Q) and expanded (50Q and 94Q) polyglutamine in N2a cells using the *Mbnl1* minigene. In (**A**–**C**), bar charts indicate the fraction of exon inclusion (*Mbnl1* and *Clcn1*) or the inclusion of the smooth muscle (SM) exon (*Actn1*). Error bars represent the mean ± SD (*n* = 3); a two-tailed Welch’s *t*-test was used for (**A**,**B**), while Tukey’s test was applied for (**C**). The original images of gel can be found in the Supplementary Materials.

The splicing regulatory ability of ATXN1 was examined using splicing reporter constructs (minigenes) known to undergo alternative splicing. EGFP or EGFP-fused ATXN1 (27Q) was transiently overexpressed in Neuro2a (N2a) cells alongside *Mbnl1*, *Clcn1*, and *Actn1* minigenes. The splicing patterns of all three minigenes were significantly altered upon ATXN1 overexpression (Figure 1A). For *Actn1*, the usage of mutually exclusive exons was affected (Figure 1A). Similar changes in splicing regulation by ATXN1 were observed in HEK293 cells (Supplementary Figure S1A).

Next, we examined whether splicing regulatory activity was conserved in mouse Atxn1, which is highly homologous to human ATXN1 but lacks the polyglutamine (polyQ) region. Splicing assays revealed that the overexpression of EGFP-fused mouse Atxn1 induced changes in splicing patterns similar to those observed for human ATXN1 (Figure 1B, Supplementary Figure S2), confirming the conservation of splicing regulatory activity between ATXN1 orthologs. Hereafter, we used the *Mbnl1* minigene to evaluate the characteristics of ATXN1 in splicing regulation.

We then assessed the effect of polyQ length on the splicing regulatory capacity of ATXN1. ATXN1 with a normal-length polyQ tract (27Q) was compared to expanded polyQ variants (50Q and 94Q). A splicing assay using the *Mbnl1* minigene revealed that ATXN1 promotes the inclusion of *Mbnl1* exon 5 independently of polyQ length (Figure 1C). Thus, both normal-length and mutant ATXN1 possess splicing regulatory ability.

### 3.2. Multiple Regions of ATXN1 Are Involved in Its Splicing Regulatory Activity

**Figure 2:**
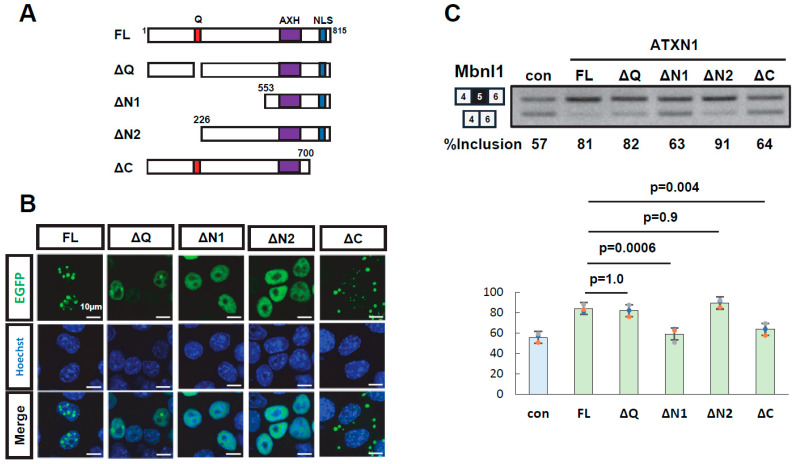
Deletion analysis of ATXN1. (**A**) Schematic diagram of full-length ATXN1 and its deletion mutants. Amino acid numbering is based on ATXN1 harboring 27Q with two histidine insertions (815 amino acids in total). (**B**) Intracellular localization of EGFP-ATXN1 mutants in Neuro2a cells. Scale bar: 10 µm. (**C**) Splicing assay results of EGFP-fused ATXN1 deletion mutants using the *Mbnl1* minigene. EGFP was used as a control (con). Bar charts indicate the fraction of exon 5 inclusion. Error bars represent mean ± SD (*n* = 3, Tukey’s test). The original images of gel can be found in the Supplementary Materials.

To identify domains required for splicing regulation, deletion mutants of EGFP-ATXN1 were generated: ΔQ (polyQ deletion), ΔN1 and ΔN2 (N-terminal deletions), and ΔC (C-terminal deletion) (Figure 2A). Protein expression and the intracellular localization of all mutants were confirmed (Figure 2B, Supplementary Figure S3). These deletion mutants were transiently expressed alongside the *Mbnl1* minigene to evaluate their splicing regulatory activity. The ΔQ mutant exhibited a splicing regulatory activity indistinguishable from that of wild-type ATXN1, demonstrating that polyQ is dispensable for this function. The ΔN1 mutant, which harbors a large deletion in the N-terminal region, exhibited a splicing pattern similar to that of EGFP alone, indicating a loss of splicing regulatory ability. In contrast, ΔN2, which contains a shorter N-terminal deletion, retained splicing regulation comparable to that of full-length ATXN1. This suggests that the central region encompassing amino acid residues 226–552 is critical for splicing regulation. Finally, the ΔC mutant, which lacks the C-terminal region, also exhibited reduced splicing regulatory ability, indicating that the C-terminal region (700–816) contributes to splicing control. Collectively, these results indicate that both the middle region (226–552) and the C-terminal region (700–816) of ATXN1 are required for splicing regulation.

### 3.3. Identification of Splicing Regulatory Regions of ATXN1

To determine the specific regions of ATXN1 involved in its splicing regulatory activity, we first examined the C-terminal region (residues 700–816), which contains a nuclear localization signal (NLS). Since splicing occurs in the nucleus, the NLS may facilitate ATXN1-mediated splicing regulation. However, other sequences within the C-terminal region may also contribute to splicing regulation independently of the NLS.

**Figure 3:**
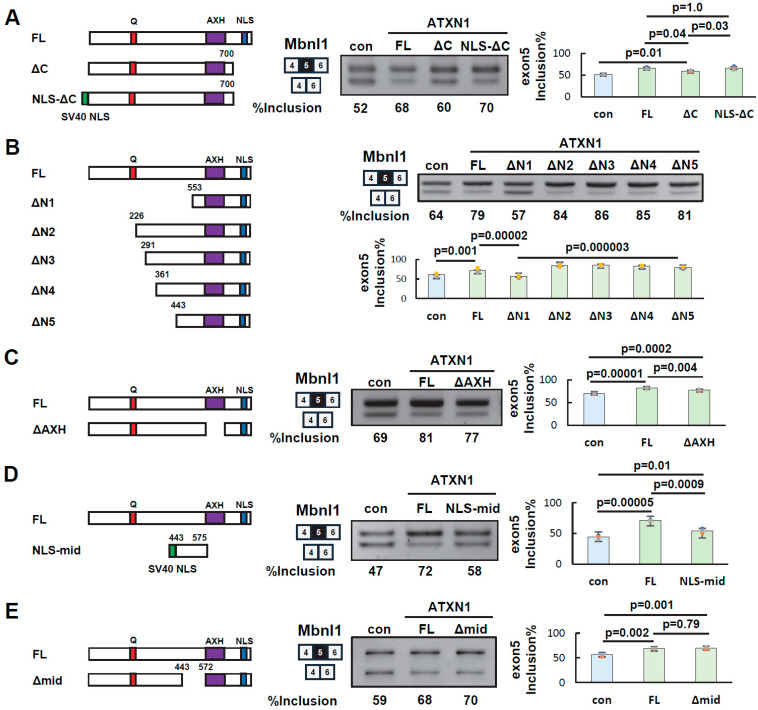
Identification of ATXN1 protein regions involved in splicing regulation. (**A**) Splicing assay of EGFP-ATXN1 mutants lacking the C-terminal region in Neuro2a cells. NLS-ΔC contains three copies of the SV40 nuclear localization signal (NLS) added to the N-terminus of the ΔC mutant. Amino acid numbering is the same as in Figure 2A. Bar charts indicate the fraction of exon 5 inclusion (*n* = 3, Tukey’s test). (**B**) Splicing assay of EGFP-ATXN1 mutants lacking N-terminal regions to determine the contribution of the middle region. Splicing assay results are shown on the right. The bar graph on the right represents the results of the splicing assay (*n* = 4, Tukey’s test). (**C**) Splicing assay of an EGFP-ATXN1 mutant lacking the AXH domain (ΔAXH). Splicing assay results are shown on the right (*n* = 3, Tukey’s test). (**D**) Splicing assay of EGFP-ATXN1 mutants containing the middle region and an artificial NLS (NLS-mid). Splicing assay results are shown on the right (*n* = 3, Tukey’s test). (**E**) Splicing assay of an EGFP-ATXN1 mutant lacking the middle region (Δmid). Splicing assay results are shown on the right (*n* = 3, Tukey’s test). In all panels, the error bars represent the mean ± SD. The original images of gel can be found in the Supplementary Materials.

To investigate this, we generated the NLS-ΔC mutant by adding an artificial NLS (three copies of the SV40 NLS) to the N-terminus of ΔC. Splicing assays showed that NLS-ΔC restored exon 5 inclusion at a level comparable to that of full-length ATXN1 (FL) (Figure 3A). These results indicate that the role of the C-terminal region in splicing regulation can be substituted by an artificial NLS. Thus, nuclear localization of ATXN1 via the NLS appears to be the key function of the C-terminal region, while other sequences in this region are not essential for splicing regulation.

Next, new deletion mutants (ΔN3, ΔN4, and ΔN5) were generated to further examine the central region (residues 226–553) in detail. As shown above, the inclusion rate of *Mbnl1* exon 5 in ΔN1 was comparable to that in the negative control (Figure 3B). In contrast, ΔN5 exhibited an inclusion rate similar to that of FL (Figure 3B). Comparable results were obtained when these mutants were tested in HEK293 cells (Supplementary Figure S1B). These findings suggest that the central amino acid residues 443–552, which differentiate these two mutants, are crucial for splicing regulation.

The AXH domain has been reported to interact with itself, other proteins, and RNA [11,18,28]. However, as shown above, the ΔN1 mutant, which retains both the AXH domain and the C-terminal NLS, did not exhibit splicing regulatory activity (Figure 3B), indicating that these domains alone are insufficient for this function. To further investigate the role of the AXH domain, we examined the ΔAXH mutant in a splicing assay (Figure 3C). Splicing assays demonstrated that ΔAXH exhibited an intermediate inclusion rate of *Mbnl1* exon 5 between that of FL and the control, suggesting that the AXH domain plays a partial role in ATXN1’s splicing regulatory function.

Finally, we evaluated the contribution of the middle region. A mutant (NLS-mid) was generated by adding an artificial NLS to a fragment of the middle region (amino acids 443–575, Figure 3D). Notably, splicing assays revealed that NLS-mid had a weak but statistically significant effect on exon 5 inclusion (Figure 3D), suggesting that the middle region alone possesses some splicing regulatory activity. We also examined the Δmid mutant, which lacks amino acids 443–572, corresponding to most of the middle region. This mutant exhibited a splicing pattern similar to that of FL (Figure 3E). Thus, at least one of the AXH domains or the middle region appears to be required for full splicing regulation by ATXN1. However, the ΔN1 and NLS-mid mutants reveal that neither domain alone is sufficient, even in the presence of an NLS.

### 3.4. Binding of ATXN1 Protein to RNA

**Figure 4:**
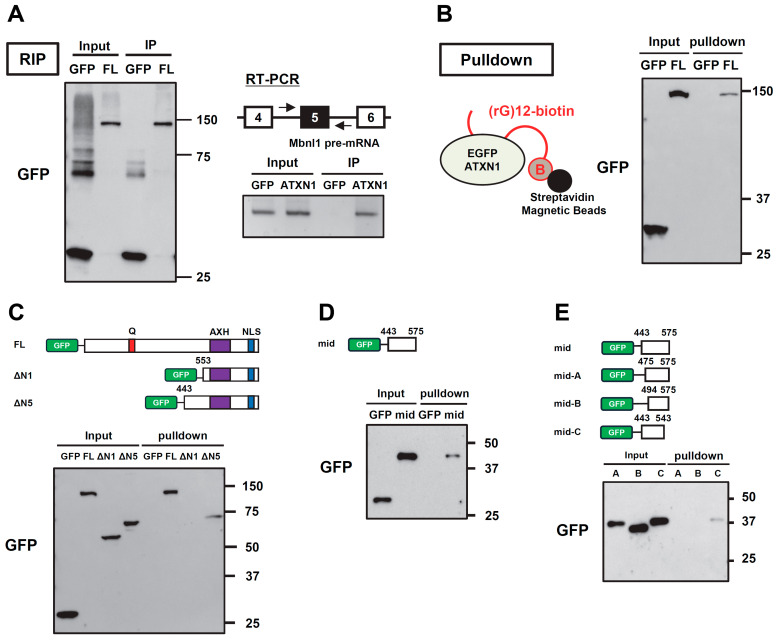
RNA binding of ATXN1 and its deletion mutants. (**A**) Ribonucleoprotein immunoprecipitation (RIP) analysis of ATXN1. EGFP (con) and EGFP-ATXN1 were introduced into Neuro2a cells. After paraformaldehyde fixation, proteins were immunoprecipitated using an anti-GFP antibody. The left panel shows Western blot results of the immunoprecipitation. The right panel shows Mbnl1 pre-mRNA detected by RT-PCR using RNA retrieved from the immunoprecipitates. (**B**) RNA pulldown assay of full-length ATXN1. EGFP or EGFP-ATXN1 was transfected into N2a cells. The cell lysates were subjected to RNA pulldown assay using biotinylated poly(rG) RNA (12-mer of guanosine) bound to streptavidin-coated magnetic beads. Input and pulldown fractions were analyzed by Western blot using an anti-GFP antibody. (**C**) RNA pulldown assay of ATXN1 N-terminal deletion mutants (ΔN1 and ΔN5), conducted as in (**B**). (**D**) RNA pulldown assay of the middle region of ATXN1. The mid mutant was analyzed using the RNA pulldown assay, conducted as in (**B**). (**E**) RNA pulldown assay of the middle region of ATXN1. We examined truncated mutants shorter than the mid variant using an RNA pull-down assay, conducted as in (**B**). The original images of gel can be found in the Supplementary Materials.

Since we found that ATXN1 regulates the splicing of the *Mbnl1* minigene, we performed RNA immunoprecipitation (RIP) to investigate whether ATXN1 binds to *Mbnl1* pre-mRNA. First, immunoprecipitation was conducted using an anti-GFP antibody to confirm the recovery of EGFP (control) and EGFP-ATXN1 (Figure 4A). RNA was then extracted from the immunoprecipitates and analyzed by RT-PCR using primers targeting *Mbnl1* intron 4 and intron 5. This analysis revealed that ATXN1 binds to *Mbnl1* pre-mRNA (Figure 4A). We also examined RIP-seq data (SRX9766718) from a recent study that comprehensively identified RNA molecules bound to ATXN1 [27]. This dataset detected multiple reads around exon 5 of *MBNL1* mRNA, which is consistent with ATXN1 binding to an RNA sequence near exon 5 (Supplementary Figure S4).

To determine which region of ATXN1 is responsible for RNA binding, we performed RNA pull-down assays. A previous study reported that ATXN1 binds preferentially to poly(rG) RNA, with the C-terminal portion of ATXN1 (residues 277–767) contributing to RNA binding [17]. First, we expressed EGFP or EGFP-ATXN1 in N2a cells, and the cell lysates were mixed with 3′-biotinylated poly(rG) RNA (12-mer of guanosine) immobilized on streptavidin-conjugated magnetic beads. After washing, the proteins remaining on the beads were detected by Western blot analysis. We detected EGFP-ATXN1, but not EGFP, in the bound fraction (Figure 4B). To further investigate the RNA-binding region of ATXN1, we tested ΔN1 and ΔN5, both of which lack the N-terminal region. A pull-down assay using poly(rG) RNA detected ΔN5, but not ΔN1, in the bound fraction (Figure 4C), demonstrating that the middle region of ATXN1 (residues 443–552) is essential for RNA binding.

Finally, we attempted to determine the minimal region required for RNA binding. The previous results suggested that the 443–575 region of ATXN1 exhibits partial splicing regulatory activity (NLS-mid, Figure 3D). To evaluate whether this region is also involved in RNA binding, we tested the 443–575 fragment (mid) in an RNA pull-down assay and confirmed its binding to RNA (Figure 4D), suggesting that this region contributes to splicing regulation through RNA binding. To further refine the RNA-binding region, we tested shorter deletion mutants of the middle region (Figure 4E). Interestingly, we detected the RNA binding of the mid-C mutant, which contains residues 443–543, whereas mutants lacking residues 443–475 did not exhibit RNA binding (Figure 4E). Thus, we identified a new protein region in the middle of ATXN1 that mediates both splicing regulation and RNA binding.

## 4. Discussion

In this study, we provide the first evidence that ATXN1 exhibits splicing regulatory activity and identify the protein regions involved in this function. We demonstrate that the splicing regulatory activity of ATXN1 is independent of the length of the polyglutamine tract. We also show that the splicing regulatory activity of ATXN1 is conserved in mouse Atxn1. To determine the domains necessary for splicing regulation, we generated deletion mutants of ATXN1. As a result, we found that the nuclear localization signals (NLSs) near the C-terminus, the AXH domain, and the middle region are important for splicing regulation. While the AXH domain is not sufficient for splicing regulation, it is necessary for full activity (Figure 3B,C). In contrast, the region exhibited some splicing regulatory activity on its own, but the deletion of this region did not affect splicing regulation (Figure 3D,E). These results suggest that the AXH domain and the middle region act redundantly, with at least one of these regions being necessary for splicing regulation in the context of the full-length protein. ATXN1 interacts with RBM17 through a linear motif overlapping with the NLS in the presence of phosphorylation at Ser776 [29]. Our results exclude the involvement of RBM17 in the regulation of *Mbnl1* by ATXN1, as the NLS-ΔC mutant, which lacks the linear motif, still retains splicing regulatory activity (Figure 3A). Besides RBM17, ATXN1 interacts with several splicing factors, such as RBFOX1 [30], U2AF65 [31], and RBPMS [32]. It is possible that ATXN1 regulates splicing through interactions with these splicing factors.

**Figure 5:**
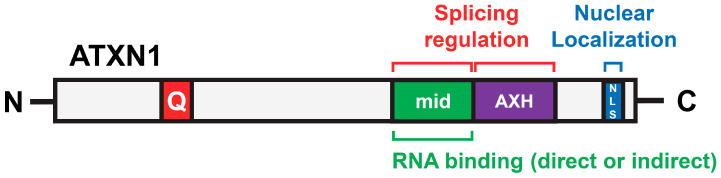
Protein regions of ATXN1 involved in splicing regulation. Three regions—the middle region (443–575), the AXH domain, and the C-terminal NLS—are essential for the full splicing activity of ATXN1. Additionally, the middle region contributes to RNA binding.

This study also provides novel insights into the RNA-binding ability of ATXN1. We performed RNA pulldown experiments using ATXN1 deletion mutants and poly(rG) RNA. Previously, a broad region of ATXN1 (residues 277–767) was reported to bind poly(rG) [17]. The AXH domain, located within this region, has been shown to exhibit poly(rG)-binding activity [18]. However, to our surprise, we failed to detect binding between poly(rG) and the ΔN1 mutant, which contains the AXH domain (Figure 4C). Instead, the mid mutant, which includes amino acids 443–575 and is distinct from the AXH domain, exhibited RNA-binding ability (Figure 4D). To the best of our knowledge, this region does not contain any previously identified RNA-binding motifs. Instead, it may harbor a novel RNA-binding motif or mediate RNA interactions indirectly through associations with other RNA-binding proteins. Based on these findings, we propose a model in which the middle region and the AXH domain of ATXN1 cooperatively or redundantly interact with target RNA and/or associated proteins to regulate splicing, with NLS-mediated nuclear localization enhancing the efficiency of this regulation (Figure 5).

Although the exact ATXN1 binding site on the *Mbnl1* minigene remains unclear, RIP experiments suggest that it binds near exon 5. Interestingly, previously published RIP-seq data also confirm that ATXN1 interacts with sequences near exon 5 of endogenous *MBNL1* mRNA [27]. These findings imply that ATXN1 may remain bound to mRNA after splicing and contribute to RNA metabolism beyond splicing. Future studies will be required to identify endogenous pre-mRNAs targeted by ATXN1. Apart from splicing, ATXN1 exhibits concentration-dependent phase separation, a process involving both self-association and interactions with RNA [33]. Additionally, ATXN1-containing inclusion bodies have been found to contain RNA [34]. Therefore, the RNA-binding property of ATXN1 is crucial for understanding inclusion body formation and the dysregulation of gene expression observed in disease.

## 5. Conclusions

This study provides the first direct evidence of the splicing regulatory activity of ATXN1. By utilizing deletion mutants, we successfully identified functional domains involved in splicing regulation. Furthermore, we discovered a novel region that mediates RNA binding. The findings of this study support further detailed investigations into RNA metabolism in ATXN1-related diseases.

## Data Availability

The original contributions presented in this study are included in the article/Supplementary Materials. Further inquiries can be directed to the corresponding author.

## Supplementary Materials

The following supporting information can be downloaded at https://www.mdpi.com/article/10.3390/biom15060782/s1. Table S1: List of oligonucleotides used in this study; Table S2: List of antibodies used in this study; Figure S1: Splicing assay of ATXN1 in HEK293 cells; Figure S2: Splicing assay of mouse Atxn1 in Neuro2a cells; Figure S3: Expression of EGFP-fused ATXN1 mutants; Figure S4: Association of ATXN1 with *MBNL1* transcripts in a RIP-seq analysis; Figure S5: Splicing assay of *Mbnl1* using polyacrylamide gels; Figure S6: Uncropped original images.

## Abbreviations

The following abbreviations are used in this manuscript:

AXH	ATXN1-HBP1
EGFP	enhanced green fluorescence protein
NLS	nuclear localization signal
polyQ	polyglutamine
RIP	RNA immunoprecipitation
SCA1	spinocerebellar ataxia type 1
