# Supplementary material for: Identification of Splicing Regulatory Activity of ATXN1 and Its Associated Domains
Source: Biomolecules. 2025 May 28;15(6):782. doi: 10.3390/biom15060782 (PMC12191242; doi:10.3390/biom15060782)
Supplement: Supplementary file 1 [file biomolecules-15-00782-s001.zip › biomolecules-3626058-supplementary.pdf]

Supplementary Materials

Identification of the Splicing Regulatory Activity of ATXN1 and Its Associated Domains

Ai Ohki[1], Masahide Kato[1], Yoshitaka Aoki[1], Arisa Kubokawa[1], Motoaki Yanaizu[1], Yoshihiro Kino[1]
[1]Department of RNA Pathobiology and Therapeutics, Meiji Pharmaceutical University, 2-522-1, Noshio, Kiyose-shi, Tokyo 204-8588, Japan
[*]Corresponding author: Tel.: +81-42-495-8679, E-mail: kino@my-pharm.ac.jp

Table S1: List of oligonucleotides used in this study
Table S2: List of antibodies used in this study
Figure S1: Splicing Assay of ATXN1 in HEK293 Cells
Figure S2: Splicing Assay of Mouse Atxn1 in Neuro2a Cells
Figure S3: Expression of EGFP-Fused ATXN1 Mutants
Figure S4: Association of ATXN1 with *MBNL1* Transcripts in a RIP-Seq Analysis
Figure S5: Splicing Assay of *Mbnl1* Using Polyacrylamide Gels
Figure S6: Uncropped Original Images

Table S1. List of oligonucleotides used in this study

Primer for construction

construct	primer	Sequence(5' to 3')
ATXN1	nest-ATXN1-Fw	cagagccatccagacagtgaaacagtcacc
	nest-ATXN1-Rv	agagccacgtttcctttcccccacgctgcc
	BamHI-ATXN1-Fw	aaaaagatctatgcaatacgccgactccggcagcc
	XhoI-ATXN1-Rv	aaactcgagcttgcctacattagaccggccttc
Atxn1 (mouse)	nest-Atxn1-Fw	accagagccggccagccagtgaaacagccaccgtg
	nest-Atxn1-Rv	ctacagtacagtaatctggatacagacagtaaaag
	BamHI-Atxn1-Fw	aaaaggatccatgaaatccaaccaagagcggagca
	SalI-Atxn1-Rv	aaaaagtcgacctacttgcccacgttagatcggcc

Primer sets used for splicing assay

Target	primer	Sequence (5' to 3')
mouse Mbnl1 minigene	EGFP-C1-Fw	catggtcctgctggagttcgtg
	Mbnl1-splicing-ex-6-Rv	ggcaatgggggaagtacagcttgaggaa
rat Actn1 minigene	EGFP-C1-Fw	catggtcctgctggagttcgtg
	Actn1-splicing-Rv	gtcgaccttatctccagccaggatcttgaa
mouse Clcn1 minigene	EGFP-C1-Fw	catggtcctgctggagttcgtg
	Clcn1-minigene-Rv	gtcgacctccaagtggtgttccaaaacagc

RT-PCR primers for RIP

Target	primer	Sequence (5' to 3')
Mbnl1	Mbnl1-int4-Fw	cactgctgcccccatgatgcacctctgcttgct
	Mbnl1-int5-Rv	aacaggaaaaggattatatctatttcaatcagt

Table S2. List of antibodies used in this study

primary antibodies	Source	Identifer
GFP tag Monoclonal antibody (1E10H7)	proteintech	66002-Ig
Goat Anti-Mouse IgG H&L(HRP)	abcam	ab97040

Figure S1

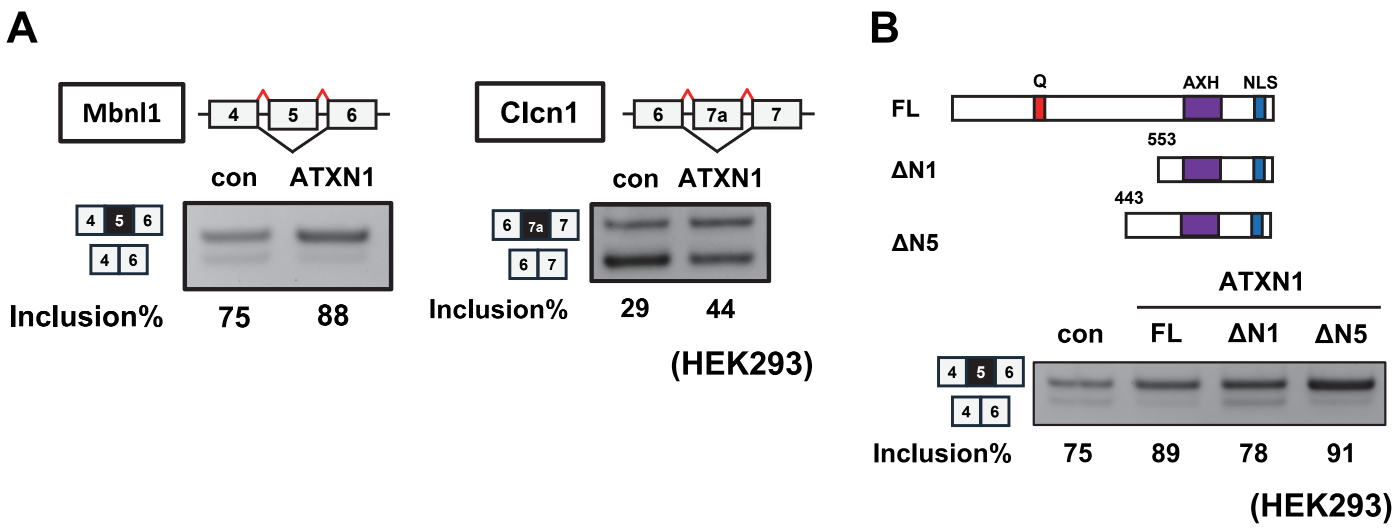

Supplementary Fig. S1. Splicing Assay of ATXN1 in HEK293 Cells
(A) Splicing assay of ATXN1 in HEK293 cells using *Mbnl1* and *Clcn1* minigenes.
(B) Splicing assay of ATXN1 deletion mutants in HEK293 cells. The splicing regulatory activity of two deletion mutants (ΔN1 and ΔN5, Fig. 3B) was tested using the *Mbnl1* minigene.

Figure S2

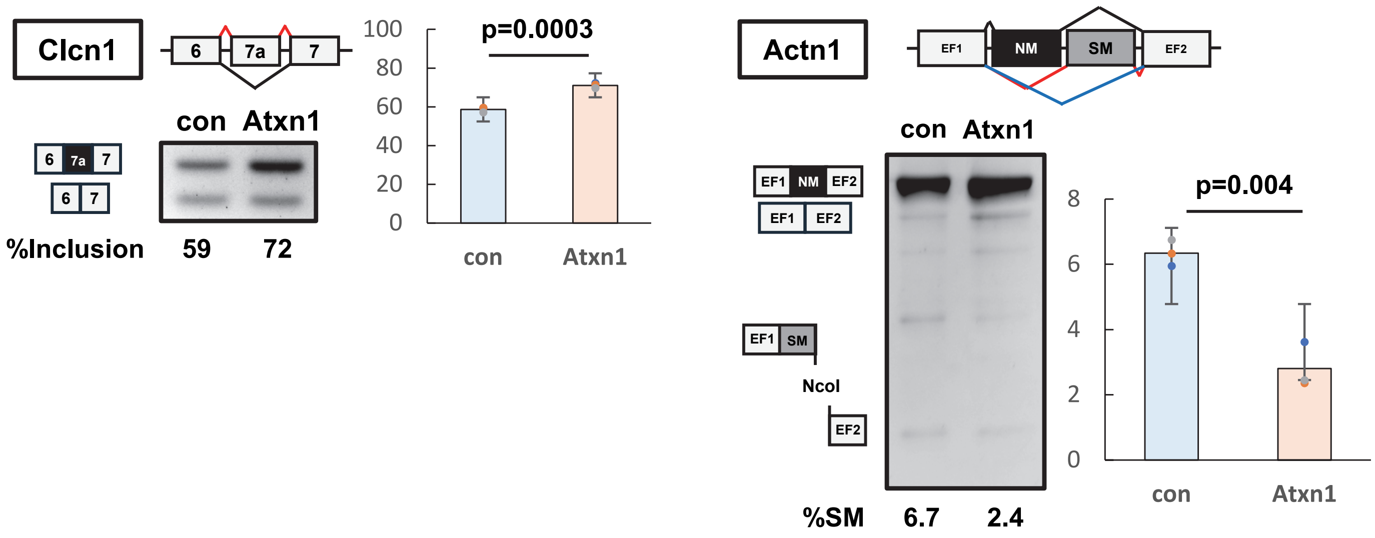

Supplementary Fig. S2. Splicing Assay of Mouse Atxn1 in Neuro2a Cells
Splicing assay of EGFP-fused mouse Atxn1 in Neuro2a cells using the *Clcn1* and *Actn1* minigenes. For *Actn1*, the PCR product was digested with NcoI to detect the inclusion of the SM exon.

Figure S3

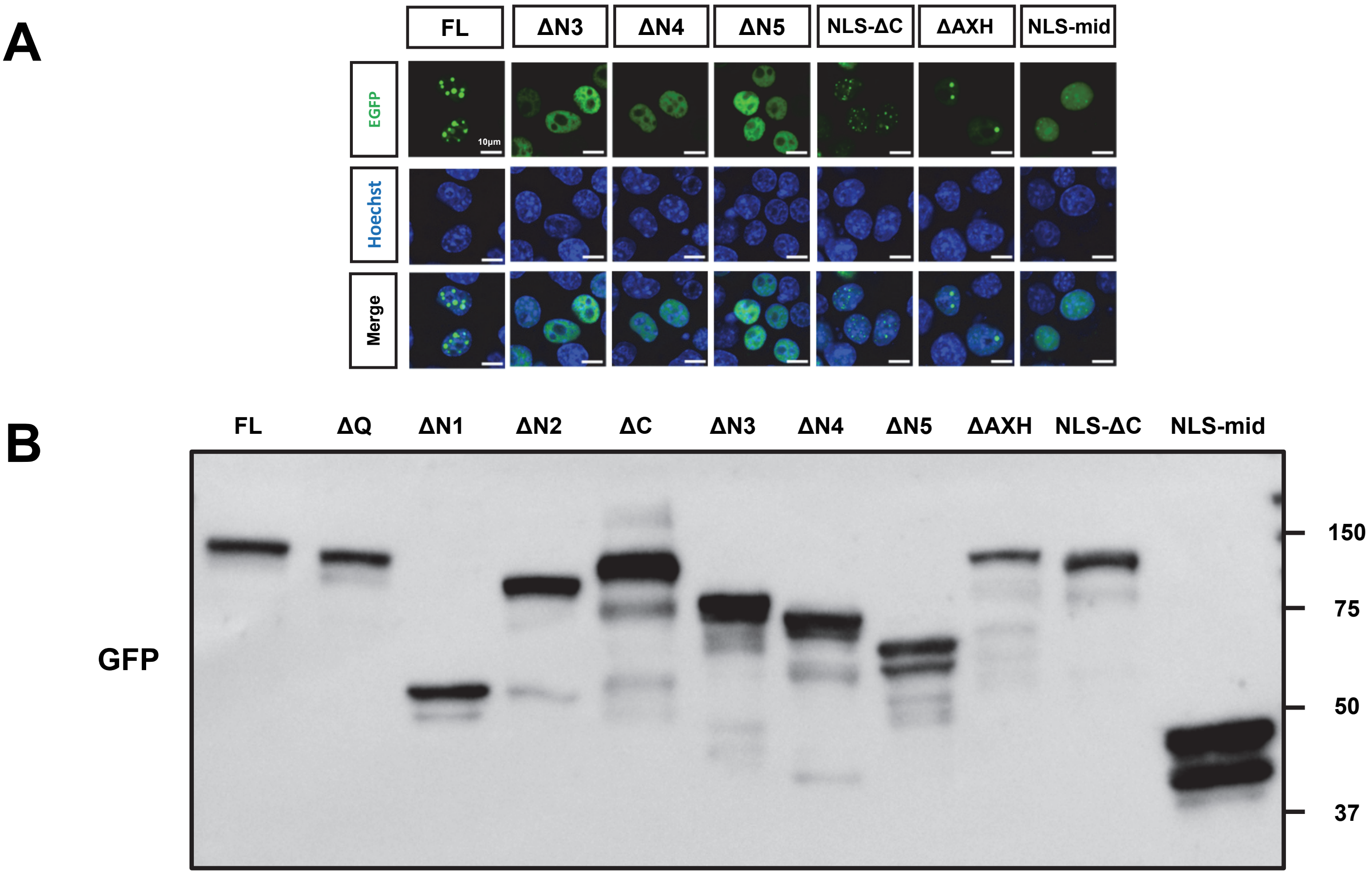

Supplementary Fig. S3. Expression of EGFP-Fused ATXN1 Mutants

(A) Intracellular localization of ATXN1 deletion mutants in Neuro2a cells. Fluorescence of EGFP and Hoechst 33342 was detected using confocal microscopy. Scale bar: 10 μm.

(B) Western blot analysis of ATXN1 mutants expressed in Neuro2a cells, detected using an anti-GFP antibody.

Figure S4

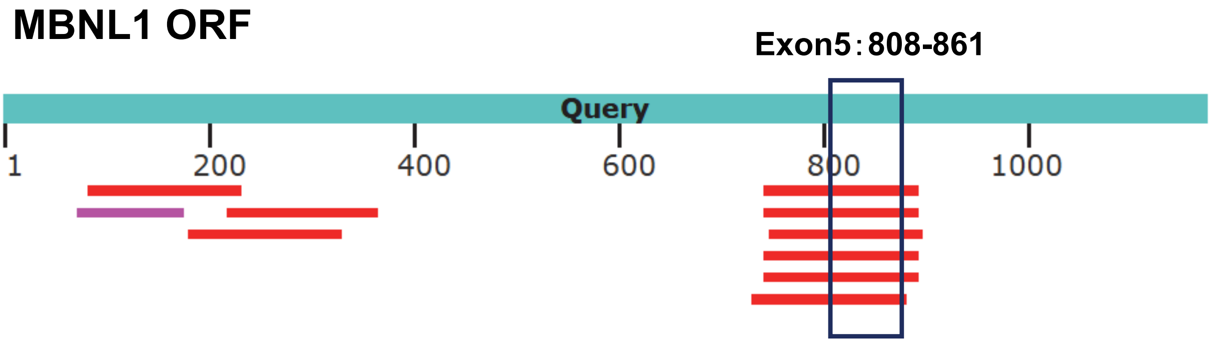

Supplementary Fig. S4. Association of ATXN1 with *MBNL1* Transcripts in a RIP-Seq Analysis
Sequence reads from a previous RIP-seq analysis (SRX9766718), in which HEK293T cells expressing mutant ATXN1 were mapped to *MBNL1* mRNA. The red and purple lines represent individual sequence reads. The numbers indicate nucleotide positions within the open reading frame (ORF) of the 42-kDa isoform of MBNL1. Exon 5 corresponds to positions 808–861.

Figure S5

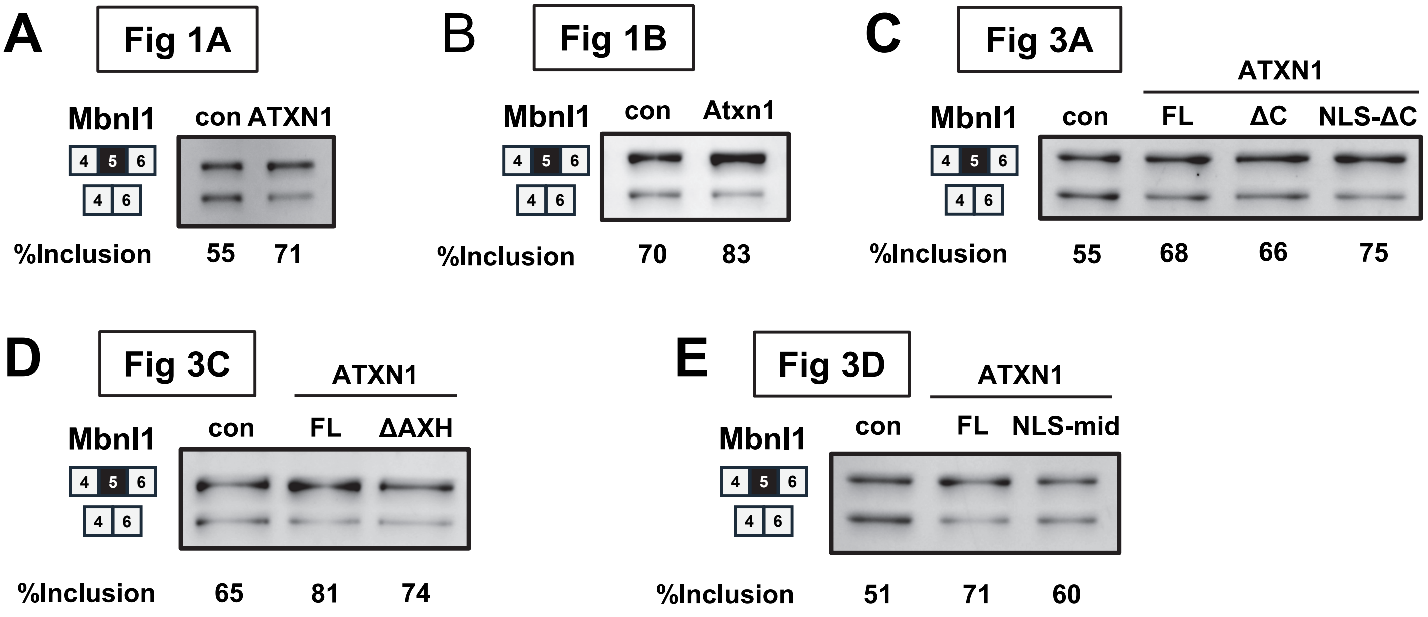

Supplementary Fig. S5. Splicing Assay of *Mbnl1* Using Polyacrylamide Gels
The results of the *Mbnl1* splicing assay, as shown in each figure, were analyzed using 12% polyacrylamide gel electrophoresis.

Figure S6

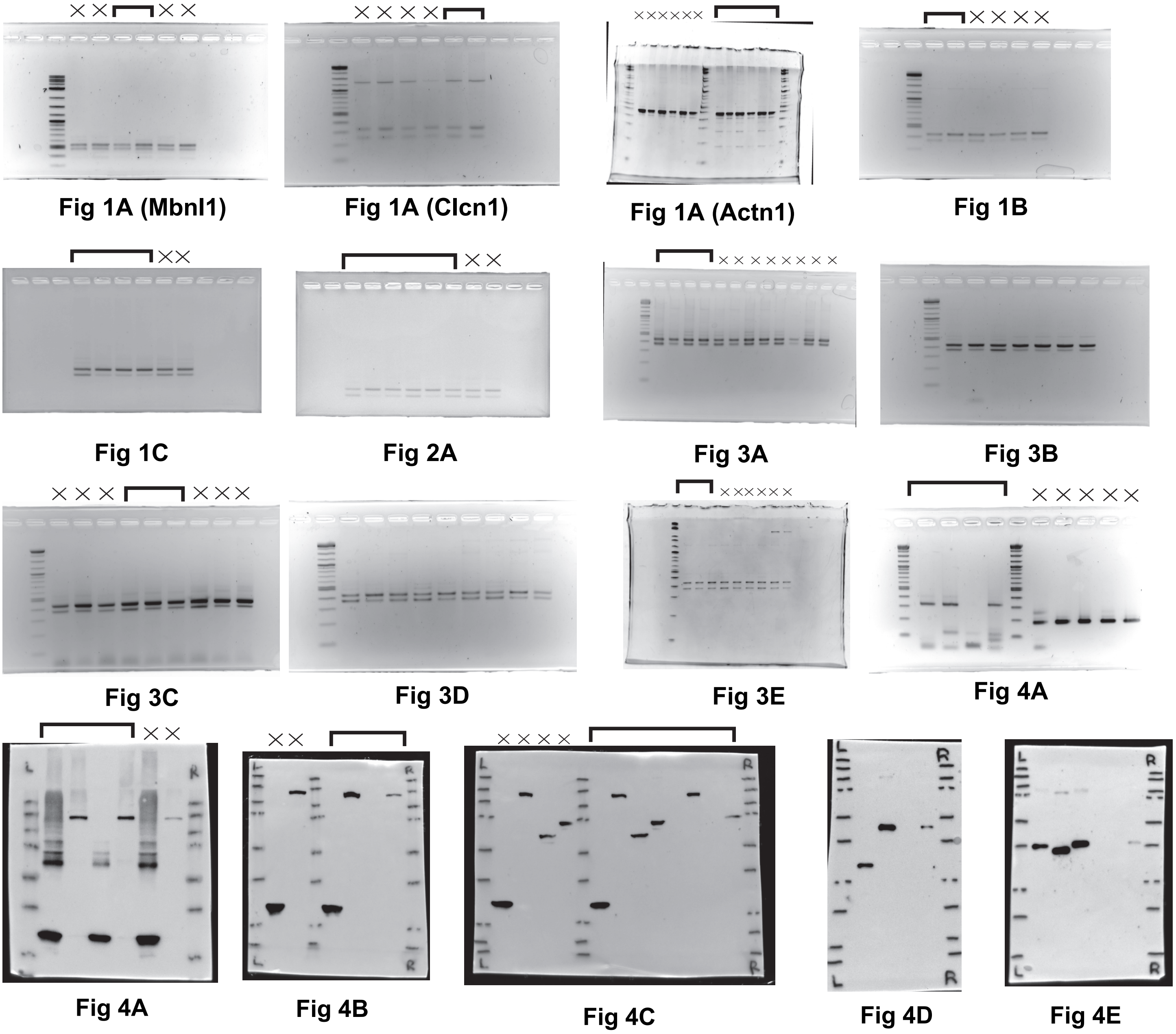

Supplementary Fig. S6. Uncropped Original Images

Figure S6 (continued)

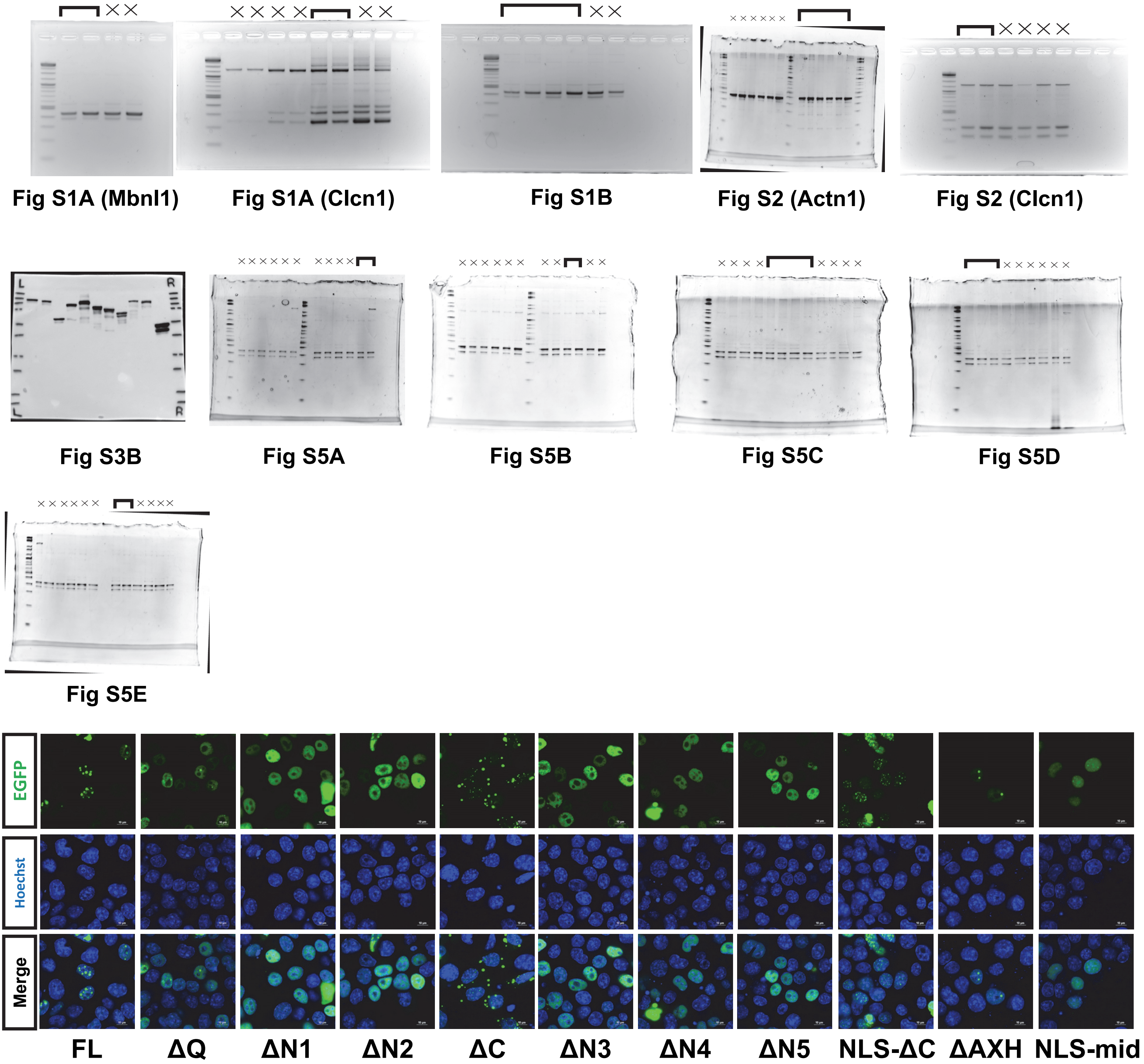

Supplementary Fig. S6. Uncropped Original Images (continued)
